# Efficacy of erlotinib in patients with relapsed gliobastoma multiforme who expressed EGFRVIII and PTEN determined by immunohistochemistry

**DOI:** 10.1007/s11060-013-1316-y

**Published:** 2013-12-19

**Authors:** Oscar Gallego, M. Cuatrecasas, M. Benavides, P. P. Segura, A. Berrocal, N. Erill, A. Colomer, M. J. Quintana, C. Balaña, M. Gil, A. Gallardo, P. Murata, A. Barnadas

**Affiliations:** 1 Medical Oncology Service, Santa Creu i Sant Pau Hospital, Sant Antoni Mº Claret nº 167, 08025 Barcelona, Spain; 2 Pathology Department, Hospital Clinic, Barcelona, Spain; 3 Medical Oncology Service, Carlos Haya Hospital, Málaga, Spain; 4 Medical Oncology Service, San Carlos University Hospital, Madrid, Spain; 5 Medical Oncology Service, Valencia General University Hospital Consortium, Valencia, Spain; 6 Althia Health, Barcelona, Spain; 7 Medical Oncology Service, Catalonian Institute of Oncology (ICO), Germans Trias i Pujol Hospital, Badalona, Barcelona Spain; 8 GEINO (Spanish Neuro-Oncology Research Group, Grupo Español de Investigación en Neuro-oncologia), Medical Oncology Service, Catalonian Institute of Oncology (ICO), L’Hospitalet de Llobregat, Barcelona Spain; 9 Pathology Department, Clinica Girona, Girona, Spain

**Keywords:** Glioblastoma (GBM), Epidermal growth factor receptor (EGFR), Progression-free survival (PFS6), Karnofsky performance status (KPS), Enzyme-inducing anti-epileptic drugs (EIAEDs), Low molecular weight heparin (LMWH), Deep vein thrombosis (DVT)

## Abstract

Epidermal growth factor receptor gene (*EGFR*) alteration is a common feature in most of glioblastoma multiforme (GBM). Robust response of anti-EGFR treatments has been mostly associated with the *EGFR* deletion mutant variant III (*EGFRvIII*) and expression of PTEN. We have performed a prospective trial in order to confirm the efficacy of erlotinib treatment in patients with relapsed GBM who expressed EGFRvIII and PTEN. All patients included in the trial were required to be PTEN (+++), EGFR (+++) and EGFRvIII (+++) positives by immunohistochemistry. This new phase II trial enrolled 40 patients and was design to be stopped in case of fewer than two responses in the first 13 patients. Patient eligibility included histopathology criteria, radiological progression, more than 18 years old, Karnofsky performed status, KPS > 50, and adequate bone marrow and organ function. There was no limit to the number of prior treatments for relapses. No enzyme-inducing antiepileptic drugs were allowed. The primary endpoints were response and progression-free survival at 6 months (PFS6). Thirteen patients (6 men, 7 women) with recurrent GBM received erlotinib 150 mg/day. Median age was 53 years, median KPS was 80, and median prior treatments for relapses were 2. There was one partial response and three stable diseases (one at 18 months). PFS at 6 months was 20 %. Dose reduction for toxicity was not needed in any patient. Dermatitis was the main treatment-related toxicity, grade 1 in 8 patients and grade 2 in 5 patients. No grade 3 toxicity was observed. Median survival was 7 months (95 % IC 1.41–4.7). As conclusion, monotherapy with erlotinib in GBM relapses patients with high protein expression for PTEN (+++), EGFR (+++), and EGFRvlII (+++) showed low toxicity but minimal efficacy and the trial stopped.

**Electronic supplementary material:**

The online version of this article (doi:10.1007/s11060-013-1316-y) contains supplementary material, which is available to authorized users.

## Introduction

Mutations affecting the epidermal growth factor receptor (*EGFR*) expression or activity could result in cancer. Attempting to improve patient survival, inhibition of the *EGFR* pathway is an attractive therapeutic target [1–4]. EGFR activation increases cell proliferation, migration, and invasiveness, and decreases apoptosis by downstream signaling, especially via the *RAS* pathway [1–4]. Between 40 and 50 % of glioblastoma multiforme (GBM) cases carry alterations of the *EGFR*, and approximately half of these co-express the mutated variant *EGFRvIII*, which has a deletion of exons 2–7 that generates a constitutively active receptor, even in the absence of ligand binding [2]. Several small molecules and antibodies directed against EGFR has been successfully used as EGFR inhibitors and clinically tested. Erlotinib and gefitinib belong to the group of small inhibitory molecules currently use in mono or combined therapy in some cancer diseases models.

Advanced high-grade astrocytomas as GBM has a poor outcome, with a very low survival rate. Temozolomide (TMZ) an oral alkylating agent is the main therapy used for GBM treatment, although only a partial improvement on progression free survival and overall survival has been detected. Few trials have been described to benefited from erlotinib or gefitinib [2, 3] and in these studies no clear correlation has been found between drug response and EGFR expression. Mellinghoff et al. [2], however, identified two molecular events in tumor patients who could be related with a positive response to erlotinib or gefitinib: the expression of EGFRvIII, and PTEN, a tumor-suppressor protein that inhibits the phosphatidylinositol 3′ kinase signaling pathway downstream EGFR. According with these authors, coexpression of EGFRvIII and PTEN proteins, as detected by immunohistochemistry (IHC), highly correlated with clinical responses to EGFR kinase inhibitors. To test this hypothesis, we performed a phase II study of erlotinib treatment in patients with relapsed GBM.

## Materials and methods

### Patients and treatment

All patients signed an informed consent form before enrolment. All patients had recurrent GBM. The eligibility criteria were age > 18 years, life expectancy > 8 weeks, and Karnofsky performance status (KPS) ≥ 60 with histological confirmed disease. All patients were required to have pre-treatment brain magnetic resonance images (MRI) within the 14 days before therapeutic treatment, and to have been receiving a stable steroid dosage for ≥5 days. Because erlotinib is metabolized by the cytochrome P450 isoenzyme 3A4 (70 %) and CYP 1A2 (30 %), patients taking enzyme-inducing anti-epileptic drugs (EIAEDs) were not eligible. There were no limitations regarding prior relapses and prior treatments. Normal bone marrow function, adequate liver function (SGOT and bilirubin < 1.5 times times the upper limit of normality ULN), and adequate renal function (creatinine < 1.5 mg/dL) within 14 days prior to registration, was required for all patients. Women of childbearing potential and their couples had to use adequate contraception throughout the study period and for 12 weeks after its completion. The response was evaluated using the McDonald criteria.

Exclusion criteria were: GBM previously treated with anti EGFR drugs, any previous infiltrating neoplasia within the last 5 years, severe cerebral hemorrhage following the biopsy, anticonvulsant inducer/inhibitor treatment of the CYP3A4 enzymes or treatment with other drugs that interact with the metabolism of the study drug and that could not be appropriately replaced with another drug without possible interactions; pregnant or lactating women, active cardiovascular disease, hypertension not controlled by standard anti-hypertensive medications, unstable angina, congestive heart disease (NYHA grade 3–4), cardiac arrhythmia or prior myocardial infarction less than 1 year prior to inclusion. Erlotinib tablets were taken either 1 h before or 2 h after meals, in the morning. The dose was 150 mg/day on a continuous daily basis.

Patients with recurrent disease were treated at four-week (one cycle) intervals. Treatment was continued indefinitely as long as there were no unacceptable toxicities or tumor progression. No other chemotherapy was during treatment with erlotinib.

### Pre-treatment and treatment evaluation

Within 14 days prior to treatment, medical history, physical examination, brain MRI and hematology and biochemistry blood analysis were required. A complete blood count with differential and platelet counts and a comprehensive metabolic panel were performed every 4 weeks during treatment. A physical and neurological examination was performed every 4 weeks, and brain imaging every 8 weeks. Clinical response was evaluated according MacDonald Criteria.

### Evaluations during the study

Toxicities were graded according to the National Cancer Institute Common Terminology Criteria Version 3.0. Follow-up of toxicity, neurological status, and KPS was performed monthly and MRI was performed every 8 weeks, until disease progression occurred.

### Statistical methods

Overall response (OR) (defined using by Macdonald Criteria) and progression-free survival at 6 months (PFS 6 m) were considered primary endpoints. Secondary endpoints were OS and toxicity. The planned sample size was 40 (all GBM). A Simon two-stage design (response rate P0 = 15 %, P1 = 35 %, α = 0.10, β = 0.10) required at least two responses in the first 13 patients to expand to a second cohort.

Response rate, PFS-6 (recurrent MG), and OS-12 were based on the proportion of patients known to have achieved that endpoint using, the intention to treat concept. Median PFS and OS were calculated from the Kaplan–Meier curves. Time was measured as from registration date. All patients receiving per protocol treatment were included in the safety assessment. The analysis of toxicity was reported using the CTCAE v3.0.

## Results

Between February 2008 and February 2010, 13 patients from the Medical Oncology Department, Hospital Sant Creu i Sant Pau, with relapse GBM met the inclusion criteria and were recruited into the study. The Hospital Clinical Trials Advisor Committee authorized the trial. All patients gave their written informed consent. The baseline characteristics of the patients are summarized in Table 1. The correlation data between EGFRvIII and PTEN considering the IHC, FISH and RT-PCR results are described in Table 2.

**Table 1:** Patient demographics and previous chemotherapies

Patient demographics	(*N* = 14)
Males/females	7/6
Age (median, years)	53
Performance status	
ECOG 1	5
ECOG 2	5
ECOG 3	3
Previous 1st line chemotherapy	
Stupp protocol	13
Previous 2nd line chemotherapy	
CPT11+Temozolomide	3
Extended temozolomide	5
Bevacizumab+CPT11	1
Carmustine implant after 2nd surgery	2
Procarbazine+CCNU+Vincristine (PCV)	1
Previous 3rd line chemotherapy	
Bevacizumab+CPT11	3
Extended temozolomide	2

**Table 2:** Immunohistochemistry, FISH and RT-PCR results

Immunohistochemistry results															FISH AND RT-PCR RESULTS				
Sample identification			EGFR				EGFRvIII				PTEN				FISH				RT-PCR
															*EGFR*		*PTEN*		*EGFRvIII*
No.	Block identification	Diagnosis	Intensity	%	Score	Result	Intensity	%	Score	Result	Intensity	%	Score	Result	Alteration	Result	Alteration	Result	Result
1	2006B08002/2	GBM	3	80	240	High	3	95	285	High	3	75	225	High	AMP	Positive	Monosomy	Positive	Negative
2	2007B06096/3	GBM	3	70	210	High	3	75	225	High	3	70	210	High	HP	Positive	HP	Negative	Negative
3	2007B10980/3B	GBM	3	90	270	High	NA	NA	NA	High	3	80	240	High	LT–LP	Negative	WT	Negative	Negative
4	2007B06899/2	GBM	3	80	240	High	NA	NA	NA	High	3	80	240	High	AMP	Positive	WT	Negative	Positive
5	2006B002537-1	GBM	NA	NA	NA	High	3	90	270	High	2	60	120	Intermediate	AMP	Positive	Monosomy	Positive	Negative
6	2006B004788-II	GBM	NA	NA	NA	High	NA	NA	NA	High	3	90	270	High	HT–LP	Negative	WT	Negative	Negative
7	2006B09662/2ª	GBM	3	90	270	High	3	90	270	High	3	90	270	High	AMP	Positive	LOH	Positive	Positive
8	2008B08673/C	GBM	3	80	240	High	3	80	240	High	3	80	240	High	AMP	Positive	LOH	Positive	Negative
9	2009B002927-A1	GBM	3	100	300	High	3	100	300	High	3	100	300	High	LT–LP	Negative	LOH	Positive	Negative
10	2008B13459/3E	GBM	3	70	210	High	3	60	180	High	3	75	225	High	AMP	Positive	LOH	Positive	Positive
11	10-7434-1PP	GBM	NA	NA	NA	High	3	100	300	High	3	75	225	High	AMP	Positive	Monosomy	Positive	Negative
12	10-7990-2C	GBM	NA	NA	NA	High	3	80	240	High	3	90	270	High	AMP	Positive	WT	Negative	Negative
13	B-1173-09 V	GBM	NA	NA	NA	High	NA	NA	NA	High	NA	NA	NA	High	AMP	Positive	Monosomy	Positive	Positive

*GBM* glioblastoma, *NA* not available, *AMP* amplification, *HT*–*LP* high trisomy–low polysomy, *LOH* loss of heterozygosity, *LT*–*LP* low trisomy–low polysomy, *HP* high polysomy, *WT* wild type

### Treatment and dose intensity

Thirteen patients (6 men, 7 women) with recurrent GBM received 150 mg erlotinib daily. Median age was 53 years, median KPS was 80, and media number of patient prior treatments for relapses was two.

Dose reduction for toxicity was not needed in any patient. The main treatment-related toxicity was dermatitis, grade 1 in 8 patients and grade 2 in 5 patients. No grade 3 toxicity was observed. The toxicities are summarized in Table 3.

**Table 3:** Toxicities

Adverse event	Incidence	
	N (14)	Rate (%)
Dermatitis	8	57
Grade 1		
Grade 2	5	35
Grade 3	0	0
Diarrhea		
Grade 1	6	42
Grade 2	1	7

**Fig. 1:**
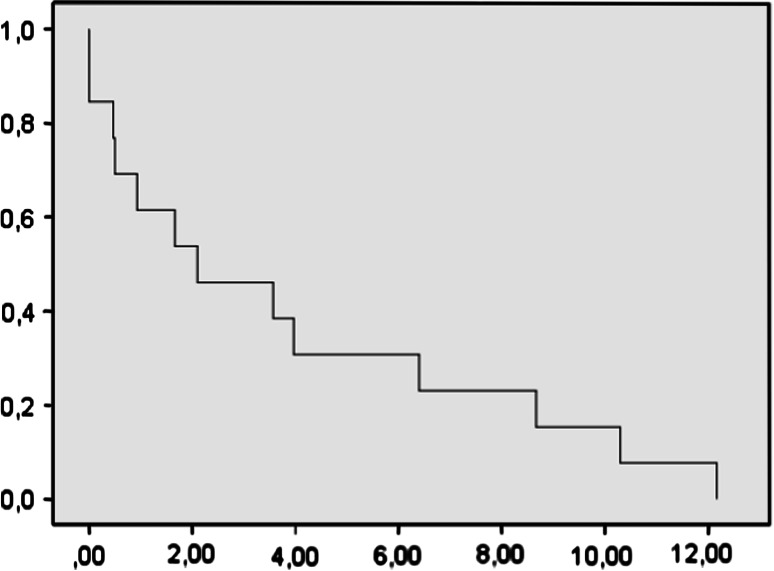
Kaplan–Meier plots of progression free survival

**Fig. 2:**
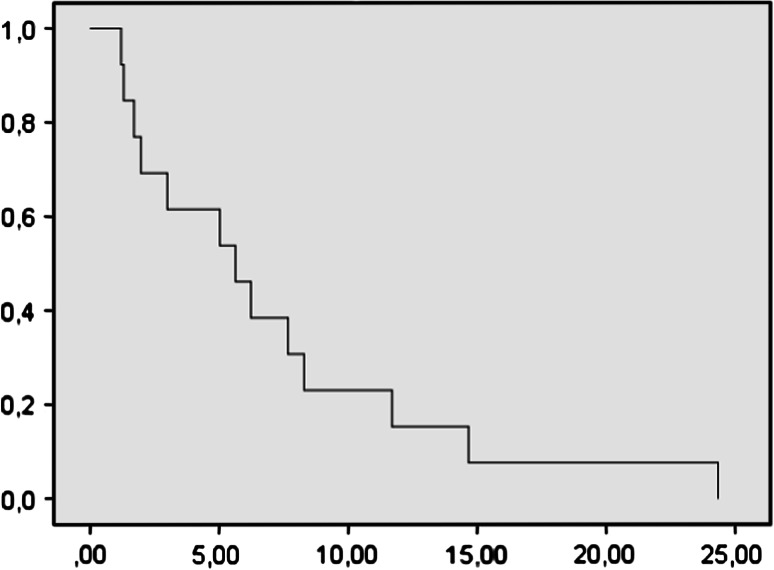
Kaplan–Meier plots of overall survival

There was one partial response and three stable diseases (one of them still stable at 18 months). PFS at 6 m was 20 %. Median progression free survival was 3.9 months (IC 1.6–6.1). Median survival was 7 months (IC 1.41–4.7) (Figs. 1, 2). Only one patient evidenced a good response. Considering these poor results, we discarded the initial hypothesis and the study was stopped because of ethical reasons (Table 4).

**Table 4:** Types of Responses

Type of response	*N*	Rate (%)
Partial response	1	7
Stable disease	3	21
Progression disease	10	72

### Biomarker analysis

A weak significant linear trend association between EGFRvIII IHC staining (high, intermediate, or low) or negative, and *EGFR* FISH (positive vs. negative), *p* = 0.035 has been found. Nevertheless, no association was found when comparing PTEN or EGFR IHC analysis versus FISH analysis.

## Discussion

The aim of the present phase II trial, was to investigate whether the coexpression of EGFRvIII and PTEN proteins, as detected by IHC, correlates with a positive clinical outcome to erlotinib, a EGFR kinase inhibitor, as previously reported [2].

The response to EGFR inhibition in relapsed glioblastoma (GBM) has been widely studied in recent years but results are non-conclusive. Different clinical trials have been developed using EGFR inhibitors in monotherapy regime or in combination with other drugs. Although, the first trial using gefitinib give raise noresponse [1], two new studies, offered encouraging results [2, 3].

Mellinghoff et al. [2] showed that coexpression of EGFRvIII and PTEN was associated with the response observed when using EGFR kinase inhibitors, suggesting that EGFRvIII and PTEN expression by IHC was sufficient to select the patient responders cohort to EGFR inhibitors. Survival obtained in these patients after the treatment with EGFR inhibitors was 21.7 months in responders versus 5.8 months in non-responders (*p* = 0.01). The average time to progression was 9.7 versus 1.7 months (*p* < 0.001). No mutations of *EGFR* gene were detected in seven patients who responded. *EGFRvIII* was detected in 46 % of patients. Six out of the 12 patients whose tumor expressed EGFRvIII responded to EGFR inhibitors (*p* = 0.003). None of the 13 patients whose tumors lacked PTEN responded to treatment. The probability of response was highest when the tumors coexpressed EGFRvIII and PTEN (OR 51; 95 % IC 4–669; *p* < 0.0001) [2].

An erlotinib therapeutic response on relapse GBM patients has been previously reported [3]. In this trial a partial responses was detected on 8 of 41 treated patients with this agent, concluding that the patients with GBM tumors who have high levels of EGFR expression and low levels of phosphorylated PKB/Akt had better response to erlotinib treatment than those with low levels of EGFR expression and high levels of phosphorylated PKB/Akt [3].

However, EGFR inhibitors response on GBM is still controversial. Despite those two studies with positive results, most studies in patients with relapsed glioblastomas treated with EGFR inhibitors obtained negative findings, such as the EORT randomized phase II trial [5]. This study included 110 patients, 54 treated with erlotinib and 56 with TMZ or BCNU (bis-chloroethylnitrosourea), showing that PFS at 6 months was 12 % for erlotinib and 24 % for the control arm and an similar OS similar in both arms. In contrast with the study of Mellingoff’s study, patients with EGFRvIII mutations [13] in the erlotinib arm and eight in the control arm) had shorter PFS and survival. Investigators concluded that response to erlotinib was not correlated with the expression of EGFR or EGFRvIII [5]. In our present study patient overall survival was only 7 months, and median progression-free survival was only of 3 months; furthermore, only one patient evidenced a good response. Considering these poor results, we stopped the study for ethical reasons. Similarly, other trials using erlotinib in first-relapse glioblastoma also stopped early on due to the low response rate. In this trial described by Young et al [6] median response, 6-month progression-free survival, and median survival were similar to those described in our study. *EGFR* amplification was never found associated with erlotinib activity. Raizer et al. [7] found similar results on 53 erlotinib treated patients with recurrent glioma with median PFS in 2 months. They concluded that erlotinib gives minimal response for recurrent GBM.

Given the poor results of EGFR inhibitors in monotherapy, several groups have developed combinatory therapy in an attempt to improve the outcomes. However, results have been discouraging. So, a pilot study to assess the tolerability and efficacy of everolimus with gefitinib in patients with recurrent GBM founding a clinical benefit in 37 % of patients, with a PFS of 2.6 months [8].

Following new combo therapy trials, EGFR inhibitors (erlotinib and/or gefitinib) were use in combination with the inhibitor of mTOR inhibitor sirolimus. In one trial, 19 % of the 28 enrolled GBM patients experienced a partial response and 50 % had stable disease, with a 6-month PFS rate of 25 %. A surprisingly positive result was obtained in an small cohort of patients [9]. Erlotinib was also combined with carboplatin on treatment of recurrent glioblastomas [10]. At this phase II study Groot et al. [10] found an average time to progression of 15.2 weeks, slightly better data than previously published, but using a low number of heterogeneous selected patients. None of the 32 recurrent glioblastoma patients achieved either complete or partial responses when erlotinib was used in combinatory therapy with sirolimus [11].

Furthermore, erlotinib was also used in combo therapy with biological therapeutic compounds as bevacizumab in a phase II study of recurrent malignant glioma tumors. Bevacizumab (10 mg/kg) was given intravenously every 2 weeks. PFS-6 and median OS were 28 % and 42 weeks for GBM patients. Most of the toxicities were mild. Unfortunately, erlotinib did not seem to add any further clinical benefit compared to patients who received bevacizumab alone.

Although the pharmacokinetics of erlotinib in both healthy volunteers and adult patients with cancer has been well characterized [12–14]. Very little is known about the central nervous system penetration and exposure to this drug which is a critical issue in the treatment of patients with primary brain tumors [15]. Vivanco et al. [16] demonstrated that the disappointing clinical activity of first-generation EGFR inhibitors in GBM versus lung cancer might be attributed to the different conformational requirements of mutant EGFR.

Regarding our study, one possible explanation for the negative results could be related with the plethora of genetic alterations found in the glioblastoma tumors [17]. Molecular analysis of these tumors identified gene *EGFR* amplification and multiple types of *EGFR* mutations, the most common being *EGFR* variant III (*EGFRvIII*), loss of the tumor-suppressor protein *PTEN*, overexpression of *PDGFR* (platelet-derived growth factor receptor) and a mutation in gene *TP53* [18].

Amplification of the EGFRvIII fragment by RT-PCR was detected in 4/13 cases (30 %), similarly to recent studies [19]. However, no correlation was found between EGFRvIII IHC and RT-PCR analysis results. No differences in IHC scoring were detected between cases harboring an *EGFRvIII* RT-PCR positive result versus those cases that did not shown the exons 2–7 deletion variants.

Since PTEN has been described as required for a response to EGFR inhibitors [20], and previous studies have shown no responses in patients whose tumors lack PTEN [2], positive expression of PTEN by IHC was considered as inclusion criteria for this study. All samples from the 13 patients were positive for PTEN by IHC. Surprisingly, when measured by FISH, using specific probes, PTEN gene copy number was altered, both by LOH or monosomy in 8 out of 13 patents (61.5 %). FISH analysis allows a reliable detection of the status of the gene but may not be a definitive reflect of the status of the protein. Moreover, the election of the antibody used for IHC analysis may also be determinant for PTEN protein status analysis [21]. In our study, we used the PTEN 6H2.1 clone (DAKO), as described in previous studies [2], on which responders almost 50 % of PTEN positive patients [2]. Studies in larger cohorts with positive response results are needed to elucidate the correct approach for PTEN status.

Moreover, a better probability of a clinical response to EGFR kinase inhibitors was associated with coexpression of EGFRvIII and PTEN [2]. In our study, a partial response was shown in one out of the tree patients showing this pattern of alterations.

We found a weak significant linear trend association between EGFRvIII IHC results (being high, medium, low or negative) and *EGFR* FISH (positive vs. negative, *p* = 0.035). Previous studies have shown that there is an association between the presence of EGFR gene amplification and the EGFR genetic variant III in GBM and other tumor types [22], being patients carrying both *EGFR* amplification and *EGFRvIII* those with a worse survival. In our studies, the four *EGFRVIII* positive patients did also shown *EGFR* amplification, being a feasible reason for the poor response obtained.

Since the published data correlating PTEN and EGFRvIII IHC status to EGFR inhibitor response in glioblastoma patients, there has not been one single study that recapitulated this data. Consequently, our results support that EGFRVIII and PTEN measurement by IHC is not a solid approach for patient selection for anti-EGFR therapy, being EGFR also a marker to be included in the selection. Our results also concluded the relevance of FISH and PCR as detection of PTEN and EGFR measurement in future trials. In conclusion, we found that erlotinib provided minimal beneficial activity on relapse GBM patients and therefore, we consider that this drug is not cost-effective in the treatment relapsed GMB patients who express EGFRVIII and PTEN as identified by IHC.

## Electronic supplementary material

Below is the link to the electronic supplementary material.

Supplementary material 1 (DOC 30 kb)

Supplementary material 2 (DOC 24 kb)
