# Supplementary material for: Efficacy of erlotinib in patients with relapsed gliobastoma multiforme who expressed EGFRVIII and PTEN determined by immunohistochemistry
Source: J Neurooncol. 2013 Dec 19;116(2):413–9. doi: 10.1007/s11060-013-1316-y (PMC3890043; doi:10.1007/s11060-013-1316-y)
Supplement: Supplementary file 1 — Supplementary material 1 (DOC 30 kb) [file 11060_2013_1316_MOESM1_ESM.doc]

**Tissue samples, immunohistochemistry (IHC) and molecular analyses**

Tumor specimens were collected from patients who underwent surgery at prior to any therapy. The samples were fixed and embedded in paraffin for routine diagnosis. All tumors were diagnosed as glioblastoma (WHO grade 4) according to the WHO grading system. Several serial sections (4 µm) were obtained from each specimen for IHC, FISH and RNA studies. Prior to analyses, an hematoxylin-eosin stained section was evaluated by a pathologist and regions of interest were identified based on histopathology and quality criteria including tumor content, appropriate fixation and necrosis.

*EGFRVIII and PTEN Immunohistochemistry*

Paraffin-embedded tissue sections underwent immunohistochemical analysis using PTEN monoclonal mouse anti-human antibody (clone 6H2.1, Ref. M362729, DAKO), wild type EGFR monoclonal mouse anti-human antibody (Clone DAK-H1-WT. Ref-M729829. DAKO), and for EGFRVIII we used the monoclonal mouse anti-human EGFRPy1197, phosphorylation site specific antibody (Ref M729929, DAKO).

Immunohistochemical evaluation and scoring was performed independently by two pathologists (MC and AG), who were unaware of the results of the molecular analysis. Scores of 0 and 1 were considered to indicate PTEN loss. If staining for PTEN was heterogeneous, tumors with diminished or absent staining in at least 25 percent of the section were considered PTEN-deficient. Tumors showing intermediate to strong immunostaining intensity for EGFR and EGFRvIII were considered positive.

Quantitative image analysis to confirm the pathologists’ scoring was also performed using Soft Imaging System software.

*EGFR and PTEN FISH analyses*

EGFR and PTEN gene copy number was investigated by FISH using the LSI EGFR/CEP 7 and LSI PTEN (10q23) / CEP 10 dual color probes for EGFR and PTEN, respectively (Vysis, Abbott Laboratories, IL), according to standardized commercial protocols. Briefly, sections were incubated at 60ºC overnight, deparaffinised and incubated in 10 mmol/L sodium citrate (pH 6.0) solution using a decloaking chamber for 20 min. After pepsin HCL digestion for 8 minutes at 37ºC, samples were dehydrated and probes were applied directly to the slides. An individual slide was used for each gene copy number evaluation. Parafilm was used for cover-slip sealing, and slides were denatured at 80ºC for 4 minutes using an automated hybridization chamber, and then incubated overnight at 37ºC. After two post-hybridization washes in 2×SSC-0.3% NP-40, pH 7.2 (2 min at room temperature and 2 min at 73ºC, respectively), slides were counterstained with DAPI I DNA stain (1000 ng/µL, Vysis). The FISH evaluation was performed by trained personnel blinded to the patients’ clinical characteristics and all other molecular variables. A Nikon immunofluorescence microscope was used. For EGFR FISH abnormalities, we followed the Cappuzzo et al. 2005 classification ^(22)^ scheme that includes 6 categories: disomy, high trisomy, low trisomy, low polysomy, high polysomy, and amplification. In this scheme both high polysomy (≥4 copies in (≥40% of cells) and amplification (≥15 copies of EGFR per cell in ≥10% of analyzed cells) were considered positive for EGFR gene amplification. Criteria for PTEN gene anomalies were defined by use of histologically normal brain specimens (data not shown). Loss of the q arm of chromosome 10 required the overall mean PTEN / CEP10 ratio to be less than 0.9. Monosomy was defined as the presence of a single copy of both PTEN and CEP10 probes in ≥10% of analyzed cells. Simple gain required 10% or more of nuclei with three or more locus-specific probe signals. As for the EGFR gene, both high polysomy (≥4 copies in ≥40% of cells) and amplification (≥15 copies of PTEN per cell in ≥10% of analyzed cells) were also considered gene copy number alterations for PTEN locus.

*RNA extraction and EGFRvIII detection*

Total RNA was extracted from paraffin-embedded tissues using the FFPE RNA kit (Qiagen) and an automated Qiacube extractor after manual macrodissection of the most tumour-rich areas from up to 10 unstained slides.

RNA were used for reverse transcription by means of the MMLV transcriptase and following standardized protocols RNA quality was tested by means of real time amplification of the ABL gene following previously described standardized methods ^(23)^. *EGFRVIII* amplification was performed using two pairs of junction-specific primers that recognize the specific sequences created by the in-frame genomic deletion within exons 2 and 7 of the EGFR variant ^(24)^. This results in amplification of two fragments of 90 bp and 131 bp by conventional RT-PCR, in a 25 µL reaction containing 3 µL of cDNA, 200 mmol/L of one of each primer pairs and 1.05 U of the Expand^TM^ PCR High Fidelity PCR System (Roche Applied Science) was performed in a GeneAmp® PCR System 9700 thermal cycler (PE Biosystems) under the following cycling conditions: denaturation at 94°C for 2 min followed by 42 cycles consisting of 94°C denaturation for 30 sec, 56°C annealing for 30 sec and 72°C extension for 1 min. Direct sequencing was used to confirm positive cases. A cell line carrying the *EGFRvIII* was used to standardize the approach and as a positive control for all batches.
