# Supplementary material for: Efficacy of erlotinib in patients with relapsed gliobastoma multiforme who expressed EGFRVIII and PTEN determined by immunohistochemistry
Source: J Neurooncol. 2013 Dec 19;116(2):413–9. doi: 10.1007/s11060-013-1316-y (PMC3890043; doi:10.1007/s11060-013-1316-y)
Supplement: Supplementary file 2 — Supplementary material 2 (DOC 24 kb) [file 11060_2013_1316_MOESM2_ESM.doc]

**REFERENCES**

1. Rich JN, Reardon DA, Peery T, Dowell JM et al. Phase II trial of gefitinib in recurrent glioblastoma. J Clin Oncol. 2004;22:133-42.

2. Mellinghoff IK, Wang MY, Vivanco I, Haas-Kogan DA et al. Molecular determinants of the response of glioblastoma to EGFR kinase inhibitors. N Engl J Med. 2005;353:2012-24.

3. Haas-Kogan DA, Prados MD, Tihan T, Eberhard DA et al. Epidermal growth factor receptor, protein kinase B/Akt, and glioma response to erlotinib. J Natl Cancer Inst.2005;97:880-7.

4. Pelloski CE, Ballman KV, Furrth AF, Zhang L et al. Epidermal growth factor receptor variant III status defines clinically distinct subtypes of glioblastoma. J Clin Oncol. 2007;25:2288-95.

5. Van den Bent MJ, Brandes A, Rampling R, Kouwenhoven MC et al. Randomized phase II trial of erlotinib (E) vs. temozolomide (TMZ) or BCNU in recurrent glioblastoma multiforme (GBM): EORTC Brain Tumor Group Study 26034. J Clin Oncol 2009;10;27:1268-74.

6. Yung WK, Vredenburgh JJ, Cloughesy TF, Nghiemphu P et al. Safety and efficacy of erlotinib in first-relapse glioblastoma: a phase II open-label study. Neuro Oncol. 2010;12:1061-70.

7. Jeffrey J. Raizer, Lauren E, Andrew B, Chang S.M. et al. A phase II trial of erlotinib in patients with recurrent malignant gliomas and non progressive glioblastoma multiforme postradiation therapy. J. Neuro Oncol. 2010;12:95-103.

8. Nguyen TD, Lassman AB, Lis E, N. Rosen et al. A pilot study to assess the tolerability and efficacy of RAD-001 (everolimus) with gefitinib in patients with recurrent glioblastoma multiforme (GBM). Journal of Clinical Oncology, 2006 ASCO Annual Meeting Proceedings Part I. Vol 24, No. 18S (June 20 Supplement), 2006:1507.

9. Doherty L, Gigas DC, Kesari S, Drappatz J et al. Pilot study of the combination of EGFR and mTOR inhibitors in recurrent malignant gliomas. Neurology2006;67:156-8

10. De Groot JF, Gilbert MR, Hess KR, Hess KR et al. Phase II study of combination carboplatin and erlotinib in patients with recurrent glioblastoma multiforme. J Neurooncol. 2008;90:89-97.

11. David A. Reardon, Annick Desjardins, James J, Gururangan S et al. Phase 2 trial of erlotinib plus sirolimus in adults with recurrent glioblastoma. J Neurooncol. 2010;96:219-30.

12. Sathornsumetee S, Desjardins A, Vredenburgh JJ, McLendon RE et al. Phase II trial of bevacizumab and erlotinib in patients with recurrent malignant glioma. Neuro Oncol. 2010;12:1300-10.

13. Hidalgo M, Siu LL, Nemunaitis J, et al. Phase I and pharmacologic study of OSI-774, an epidermal growth factor receptor tyrosine kinase inhibitor, in patients with advanced solid malignancies. J Clin Oncol. 2001;19:3267–79.

14. Schaiquevich P, Panetta JC, Throm S, et al. Pharmacokinetic analysis of erlotinib and OSI-420 in pediatric patients with malignant gliomas. Abstract. Nature. 2008;83:S51.

15. [Togashi Y](http://www.ncbi.nlm.nih.gov/pubmed?term=Togashi Y%5BAuthor%5D&cauthor=true&cauthor_uid=22806307), [Masago K](http://www.ncbi.nlm.nih.gov/pubmed?term=Masago K%5BAuthor%5D&cauthor=true&cauthor_uid=22806307), [Masuda S](http://www.ncbi.nlm.nih.gov/pubmed?term=Masuda S%5BAuthor%5D&cauthor=true&cauthor_uid=22806307), [Mizuno T](http://www.ncbi.nlm.nih.gov/pubmed?term=Mizuno T%5BAuthor%5D&cauthor=true&cauthor_uid=22806307) et al. Cancer. Cerebrospinal fluid concentration of gefitinib and erlotinib in patients with non-small cell lung cancer Chemother Pharmacol. 2012;70:399-405.

16. [Igor Vivanco](http://cancerdiscovery.aacrjournals.org/search?author1=Igor+Vivanco&sortspec=date&submit=Submit)[1](http://cancerdiscovery.aacrjournals.org/content/2/5/458.full#aff-1), [H. Ian Robins](http://cancerdiscovery.aacrjournals.org/search?author1=H.+Ian+Robins&sortspec=date&submit=Submit)[11](http://cancerdiscovery.aacrjournals.org/content/2/5/458.full#aff-1), [Daniel Rohle](http://cancerdiscovery.aacrjournals.org/search?author1=Daniel+Rohle&sortspec=date&submit=Submit) et al. Differential Sensitivity of Glioma- versus Lung Cancer–Specific EGFR Mutations to EGFR Kinase Inhibitors. Cancer Discovery 2012;2:458.

17. Intratumor Heterogeneity and Branched Evolution Revealed by Multiregion Sequencing. Gerlinger M, Rowan AJ, Horswell S, Larkin J et al. New Engl J Med  2012;366:883-892.

18 Brennan CW, Verhaak RG, McKenna A, Campos B, et al. TCGA Research Network. The somatic genomic landscape of glioblastoma. Cell, 2013;155:462-77.

19. Yoshimoto K, Dang J, Zhu Shaojun, Nathanson D, et al. Development of a Real-time RT-PCR assay for detecting EGFRvIII in glioblastoma samples. Clin Cancer Res 2008;14;488-93

20. Bianco R, Shin I, Ritter CA, et al. Loss of PTEN/MMAC1/TEP in EGF receptor-expressing tumor cells counteracts the anti tumor action of EGFR tyrosine kinase inhibitors. Oncogene 2003;22:2812-22.

21. Pallares J, Bussaglia E, Martínez-Guitarte JL, Dolcet X, Llobet D, Rue M, Sanchez-Verde L, Palacios J, Prat J, Matias-Guiu X. Immunohistochemical analysis of PTEN in endometrial carcinoma: a tissue microarray study with a comparison of four commercial antibodies in correlation with molecular abnormalities. Mod Pathol. 2005;18:719-27.

22. Lv S, Teugels E, Sadones J, De Brakeleer S, Duerinck J, Du Four S, Michotte A, De Grève J, Neyns B. Correlation of EGFR, IDH1 and PTEN status with the outcome of patients with recurrent glioblastoma treated in a phase II clinical trial with the EGFR-blocking monoclonal antibody cetuximab. Int J Oncol. 2012;41:1029-35.
